# Partnered Recruitment: Engaging Individuals With Lived Experience in the Recruitment of Co‐Design Participants

**DOI:** 10.1111/hex.14131

**Published:** 2024-07-04

**Authors:** Carly Whitmore, Linxi Mytkolli, Natalie Mangialardi, Jasmine Maghera, Adam Rudick, Kitty Shephard, Stephanie Zazzera, Anika Saiva, Tracy McQuire, Peter Senior, Diana Sherifali, Peter Selby

**Affiliations:** ^1^ School of Nursing McMaster University Hamilton Ontario Canada; ^2^ INTREPID Lab Centre for Addiction and Mental Health Toronto Ontario Canada; ^3^ Diabetes Action Canada Toronto Ontario Canada; ^4^ Department of Pharmacology University of Alberta Edmonton Alberta Canada; ^5^ Faculty of Medicine & Dentistry University of Alberta, Edmonton Alberta Canada; ^6^ Department of Family and Community Medicine University of Toronto Toronto Ontario Canada

**Keywords:** co‐design, diabetes, integrated care, mental health, partnered research

## Abstract

**Background:**

Young adults with type 1 diabetes (T1D) face complex health challenges, including a heightened risk for distress. To counter this distress, there is a need to develop accessible, acceptable comprehensive care solutions that integrate diabetes and mental health care to enhance self‐efficacy and counter mental health challenges in this population.

**Objective:**

To describe the engagement of individuals with lived experience of T1D and mental health challenges in the development of a recruitment strategy to support the co‐design of an innovative integrated care programme.

**Results:**

Seven individuals with lived experience formed a Partner Advisory Council (PAC) to recruit young adults (18–29 years old) living with T1D, their friends or family and health researchers and professionals in co‐design interviews (*n* = 19) and co‐design events (*n* = 12). The PAC played a key role in developing a comprehensive recruitment strategy, overcoming traditional barriers and stigmas in the design of an integrated model of care.

**Conclusion:**

Assuming the presence of mental health challenges in young adults living with T1D during recruitment had far‐reaching impacts on the development of a whole‐person and integrated diabetes and mental health care solution. The efficient recruitment of this sample provided invaluable insights into the nuanced challenges experienced by young adults with T1D, the individual skills developed in response to their mental health challenges and the ways that this understanding can shape future programming to support mental health, quality of life and well‐being. The ongoing involvement of the PAC as co‐researchers underscores the enduring impact of patient engagement in developing integrated care solutions.

**Patient or Public Contribution:**

The co‐design of the TECC‐T1D3 model was enriched by the invaluable contributions of individuals with lived experience. This included the engagement of a diverse PAC in the recruitment of participants in co‐design interviews and co‐design events. PAC members actively participated in research decision‐making with their insights informing a robust recruitment strategy. Beyond recruitment, PAC members continue to serve as co‐researchers, shaping ongoing research and actively contributing to the TECC‐T1D3 project. Six PAC members are co‐authors on this manuscript.

## 1 Introduction

Type‐1 diabetes (T1D) is a complex chronic condition that can contribute to future health complications in the short and long term, including a high prevalence of mental health challenges [1]. Individuals living with T1D, particularly young adults (18–29 years of age), must utilize a high level of resilience and adaptability to manage the burdensome demands of day‐to‐day diabetes management while simultaneously navigating the typical social, physical and emotional changes of this age stage with increasing independence [2]. Among adolescents and young adults with T1D, the prevalence of anxiety, depression [2, 3] and eating and behavioural disorders [4, 5] is higher than in their peers without diabetes. It is estimated that upwards of one‐third of young adults living with T1D will also experience one mental illness during their life [6]. In addition to diagnosable psychiatric illness, diabetes distress, defined as a range of emotional responses to both living with and managing diabetes, such as the fear of hypoglycemia is common in those living with T1D [1]. Diabetes distress and poor mental health can contribute to impaired diabetes self‐management, prevent individuals from reaching glycemic targets and increase the risk for additional diabetes complications that significantly impact quality of life [1, 7]. As a result, the experience for many young adults living with T1D is a unique juxtaposition in which they are at high risk for experiencing disparities in service delivery and health participation while simultaneously being high‐volume and high‐cost users of health and social care services [1, 8]. Considering the changes that occur during this age stage, including the transition from paediatric to adult diabetes care, there is a need to develop accessible, acceptable comprehensive care solutions that integrate diabetes and mental health care to enhance self‐efficacy and counter mental health challenges in this population. Further, these solutions should foster strong self‐care routines, a positive outlook and supportive communities among those who share similar experiences.

Despite the importance of understanding the health issues of young adults living with T1D and a desire to offer more personalized care options, there are challenges in engaging this population in health research. An understanding of the rich, varied, and complex lived experiences of this population can contribute to the development of critically necessary community‐based care solutions capable of addressing health disparities—especially for those living with co‐occurring mental health challenges. Centring the diverse voices of those with lived experience ensures programming and models of care address the need to cultivate lasting healthy habits and quality of life, while also emphasizing the importance of user‐driven solutions tailored to different lifestyles. However, even when research agendas promote and encourage the inclusion of individuals with lived experience, health researchers often experience challenges with study recruitment [9]. Historically, widespread challenges in recruitment have been attributed to several causes, including the structure of the research enterprise (e.g., how research funding is allocated and constraints on time and resources) [10]; exclusionary or further stigmatizing recruitment approaches [11]; and scepticism about research as a result of unbalanced power differentials, historical misuse of data and lack of existing relationships [11, 12]. More specifically, for health research that focuses on diabetes, mental health and young adults, recruitment challenges include misinterpretation of eligibility requirements [13], a focus on more acute health concerns [13], the impact of current mental health status [14], use of misaligned incentives [15] and a perception that the potential to engage with the research was low [14]. One solution to overcome these challenges is to further partner with individuals with lived experience in study processes, including recruitment [9], to design programmes and services aligned to this experience [16, 17]. Bridging the gap between clinical data and real‐world experiences, weaving in the voices of those with lived experience through partnership and recruitment humanizes the narratives made available to researchers and knowledge‐users, making the co‐produced knowledge more understandable and impactful [18, 19].

To this end, the purpose of this paper is to describe an intentional approach that engaged individuals with lived experience of T1D and mental health challenges as partners or co‐researchers, rather than participants in the development of a recruitment strategy to support the co‐design of an innovative integrated care programme. This paper does not report research findings per se, but rather, with these partners as co‐authors on this paper and co‐researchers with this team, the intention is to centre the voices of people with lived experience throughout this description of the recruitment approach. To illustrate the impact of the recruitment approach from partners, recruited participant data are described alongside the strategies employed, highlighting the overall impact of this recruitment and strategy. The focus of this paper reflects a response to the specific interests and described priorities expressed by these partners.

## 2 Methods

### 2.1 TECC‐T1D3 Co‐Design Project

Recognizing the need to design models of care to support the complex mental health needs of young adults also living with T1D, a multiphase mixed methods research study is ongoing, titled: Technology‐Enabled Collaborative Care for Young Adults with Type 1 Diabetes and Diabetes Distress (TECC‐T1D3). The TECC‐T1D3 model of care adapts proven approaches and strategies, incorporating the use of the Optimal Health Programme [20, 21, 22], to integrate comprehensive care. This integration attends to the person and the population, including organizational, professional and clinical service level integration in a user‐friendly design [23]. To do this, care is delivered remotely (e.g., web‐conferencing, telephone, text messaging), with the support of existing community assets, by a care coach and peer support, and directed by a virtual care team of mental health and T1D experts. The first phase of this study involved a qualitative co‐design approach with providers, researchers, individuals with lived experience and a seven‐member Partner Advisory Council (PAC).

In the co‐design phase of the project, young adults with T1D have been engaged in project planning, prototype development and the design and testing of a user‐friendly, structured and scalable mental health intervention [24, 25]. This intervention includes a digital access platform as well as content delivered in a novel and innovative integrated programme. In the development of the TECC‐T1D3 programme, semistructured interviews with young adults living with T1D and two co‐design events with providers, researchers and individuals with lived experience (e.g., those living with T1D themselves and family or friends of those with T1D) were completed. The purpose of these interviews and co‐design events was to explore barriers, challenges and strategies employed to identify, assess and support young adults living with T1D and mental health challenges, and additionally, to understand how these individuals would use or change the programme being developed. While a co‐design approach has been used since the study's inception, it was during the planning of these interviews and co‐design events that a further need for strategic and purposive recruitment of participants that reflect the diverse needs of this population was identified.

### 2.2 Establishing a PAC

In health research, patient engagement is an established approach to ensure that the voices of those being researched impact the research [26, 27, 28]. Including patients as partners and co‐researchers ensures that research reflects patient priorities, involves activities that are accessible and produces findings that are relevant and useful [29, 30, 31]. The impacts of patient engagement are far‐reaching, with evidence demonstrating the positive effect on partners, researchers, research processes and research outputs, including in chronic disease management [32, 33]. To do this, however, it is recognized that patients need to be engaged early, engaged often and engaged throughout as many stages of the research process as possible [34]. One strategy to do this is to establish PAC [26].

**Figure 1:**
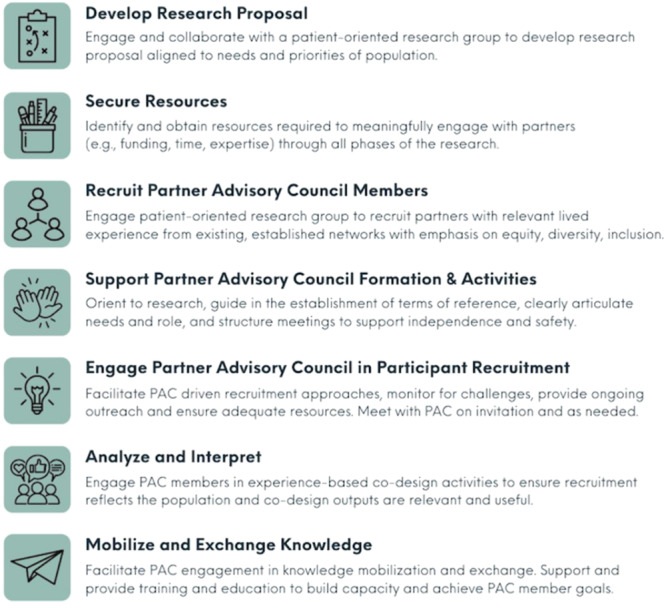
PAC engagement approach.

From early TECC‐T1D3 conceptualization work with a small group of partners, it was evident that only the voices of individuals with lived experience could authentically articulate the experiences and requirements of young adults living with diabetes. Moreover, their insights into how stigma, shame and various mental health challenges impact their care needs would be critical in refining the breadth and focus of our understanding and creating a meaningful model of care to evaluate. Partnered with Diabetes Action Canada (DAC), a Canadian Institutes of Health Research Strategy for Patient‐Oriented Research (SPOR) network member, the TECC‐T1D3 PAC was established. DAC functioned as an intermediary between the established research team and existing pockets of engaged young adults across Canada to recruit PAC members. This included outreach efforts that coincided with other DAC initiatives and engaging current partners recruited for other projects. The initial purpose of the study and details of the engagement requirements (e.g., time commitment) were shared through these groups and individuals, and young adults with lived experience could self‐identify their interest in PAC membership to the Lead of Patient Engagement with DAC (author L.M.). Careful attention was given to ensure that PAC members represented and reflected the experiences of Canadians living with T1D including a focus on sex and ethnicity. As PAC members were considered members of the research team, their demographic data were not collected as part of the research. DAC as a recognized leader in partnered diabetes research possessed the necessary knowledge and networks to lead this PAC recruitment (see Figure 1).

The purpose of this PAC was to have members serve as co‐researchers by contributing to research processes and informing research decision‐making. This included identifying gaps in understanding and contributing to user‐centric solutioning. Seven PAC members were recruited in fall 2022 from across Canada. Led by the Lead of Patient Engagement with DAC (L.M.), the PAC independently met twice a month to discuss study progress and needs, and to inform decision‐making (e.g., develop co‐design activities and interview questions, interpret and contextualize qualitative data, co‐author research publications and co‐present presentations). Meetings would start with informal discussion, experience sharing and laughter. The informality of the discussion was intentional to better support idea generation and alternative thinking. Further, this independence from the larger research team was important to permit open sharing, promote PAC cohesion, address power differentials and ensure that the PAC perceived their meeting time as safe to share their experiences and ideas. When invited to attend, bidirectional research support was provided by the co‐design lead of the project (author C.W.) including updating the PAC and the research team on discussion and decision‐making. Brainstorming and consensus activities included both structured and unstructured activities such as virtual whiteboard idea generating where PAC members individually recorded their ideas or responded to pre‐recorded questions. Consensus was achieved using activities rooted in liberating structures [35], where relational coordination and trust were built and facilitated. L.M. and C.W. both have training and experience in these methods. As PAC members were not research participants, PAC meetings were not recorded; however, notes about discussions and decisions were maintained and shared with the broader research team by authors L.M. and C.W. PAC members were compensated at a rate of $25 per hour, paid quarterly, for preparation and meeting time. Acknowledging the prevalence and burden of diabetes distress in young adults living with T1D, to support the mental health of PAC members, a distress protocol was developed and made available to PAC members. This protocol had a clear direction around escalating potential challenges to the research principal investigator and made available a curated list of relevant and free mental health resources.

## 3 Results

The PAC contributed to all decision‐making but was intensely involved in the recruitment of participants. Informed by experience, the research team, a mix of diabetes and mental health researchers and health professionals, anticipated that the recruitment of diverse co‐design participants in this study would be a challenge. For this reason, the PAC was tasked with developing a multi‐pronged recruitment strategy that was accessible, inclusive and mobilized established networks, connections and relationships. Specific to young adults, this included considerations for ‘what’ comprised participant eligibility, ‘where’ we would recruit co‐design participants and the demographic characteristics of the participants or the ‘who’ of the co‐design sample.

### 3.1 What

Participants were eligible to participate in one‐on‐one interviews if they: (1) were a young adult aged 18–29 years old; and (2) self‐identified as having a T1D diagnosis. For co‐design events, participants were eligible if they: (1) lived with T1D (i.e., did not necessarily have to be a young adult); or (2) were a friend, partner or family member of a young adult who lived with T1D; or (3) were a healthcare professional (e.g., nurse, physician or social worker) or a health researcher who worked with young adults living with T1D. Participants were not eligible if they could not participate in an interview in English language. These minimal exclusion criteria were intentionally set to achieve a highly inclusive and representative sample. In total, 19 young adult participants were recruited for the co‐design of one‐on‐one interviews and an additional 12 participants were recruited for co‐design events. These co‐design event participants included young adults with T1D (*n* = 4), friends, partners and family members of someone living with T1D (*n* = 4) and healthcare providers and researchers who work with young adults with T1D (*n* = 4).

Although TECC‐T1D3 was conceptualized as a structured mental health intervention, based on evidence of the near‐universal presence of diabetes distress in those with T1D [1], and at the recommendation of the PAC, participants were not screened for diabetes distress or other mental health challenges as an eligibility criteria. Instead, a bold and unique decision was made by the team at the behest of the PAC to simply assume the presence of mental health challenges and/or diabetes distress in all young adults living with T1D. This decision had several conceptual and methodological impacts. For PAC members, this decision was described as empowering and affirming as it acknowledged the often silent mental health impact that young adults living with T1D experience. PAC members identified that while distress and burnout may be common in this population, symptoms may not always be recognized and in including a mental health eligibility requirement, there may have been a misconception that a formal diagnosis was required to participate. Further, PAC members felt that this approach acknowledged that mental health was an ever‐fluctuating aspect of their health, particularly among those living with T1D. Rather than introducing potentially stigmatizing and exclusionary assessments as a means of having a participant prove the presence of mental health challenges, from a research perspective, it was posited that this decision would eliminate a forced or arbitrary threshold for participation, not deter individuals from participating and contribute to a shift by which the experience of these young adults is privileged over data and metrics.

While mental health data were not collected as an eligibility criterion, before their interview, participants had the option to complete the 17‐item diabetes distress scale [36] (DDS) as part of a demographic and health questionnaire. This validated tool is an objective measure of distress specifically related to living with diabetes, including T1D. The DDS asks about (1) emotional well‐being; and any distress associated with it, (2) the relationship between the individual and their physician or clinician; (3) their diabetes regimen; and (4) their interpersonal relationships. The DDS score can be used by health professionals to inform care and support [36] and has been found to be associated with other health and behavioural variables such as glycated haemoglobin [37]. A DDS score of 2.0–2.9 is considered ‘moderate distress’ while a score of > 3.0 is ‘high distress’ [36, 37]. All 19 interview participants chose to complete the DDS and on average, distress scores were considered to be moderate to high (median score 2.6, range 1.9−4.3). In completed interviews, some participants volunteered mental illness diagnoses, such as generalized anxiety disorder or depression, but this disclosure was not required or prompted, nor was the DDS score discussed with the participant.

### 3.2 Where

An intentional recruitment approach was developed through PAC brainstorming and consensus discussions with the broader research team. Capitalizing on the digital literacy of young adults, and aligned with the virtual nature of the proposed TECC‐T1D3 programme, recruitment was largely done online via PAC members and research team members alike. This included the use of social media (e.g., digital poster shared on Facebook, X—formerly Twitter), promoting the study within online communities (e.g., peer support groups, virtual communities) and distributing recruitment materials through existing listservs (e.g., organizations connected to PAC and research team members). In addition to these virtual means, recruitment was also done on a one‐on‐one basis through PAC member and research team connections. Word of mouth, or this one‐on‐one recruitment effort, was identified by the PAC as a particularly important strategy. This was because not only did the PAC describe having broad networks of individuals who would meet study eligibility, but they also knew that in this approach, the personalized and confidential nature of one‐on‐one interactions was almost like a recommendation of the study, and would enhance the likelihood of successfully recruiting participants.

Brainstorming locations of recruitment efforts included considerations for not only the geographic location of participants, as coast‐to‐coast perspectives were desired, but also for diverse perspectives and unique experiences. This included discussions around the unique facets of young adulthood, such as the experience of attending college or university, new relationships, physical activity and playing sports and transitioning from paediatric to adult care. To do this, PAC members offered insight into potential places where young adults with T1D may be as they live through these experiences (e.g., diabetes clubs at colleges and universities, or sports medicine clinics tailored to diabetes care).

We were able to engage interview participants from across regions in Canada, including Atlantic Canada (*n* = 2), Ontario (*n* = 7), Quebec (*n* = 0), Prairies (*n* = 8), Western Canada (*n* = 2) and Northern Canada (*n* = 0). Interview participants were referred to the study through all of the planned sources; however, many participants identified that their referral source was individual conversations with PAC members and research team members (*n* = 9). Otherwise, participants identified that social media (*n* = 3) or partner organizations (*n* = 7) was how they became aware of the co‐design study. Demographic data of co‐design participants were not collected as these participants included health professionals, researchers and family members of those who live with T1D.

### 3.3 Who

A matrix approach to sampling was used. As participants were recruited, disaggregated demographic data were shared with the PAC to identify gaps in the sample and to responsively adjust the recruitment approach. Demographic data of interest included age, sex and gender, ethnicity, geographic location and age at diagnosis. Selected by the PAC, these variables were ones identified to contribute to variation in experience with diabetes and, if appropriately captured, were thought to likely enhance the generalizability of the co‐designed TECC‐T1D3 programme. Adjustments to the recruitment approach included oversampling from certain locations and reaching out to known contacts with access to the desired sample. For example, after identifying that the first nine participants reported their sex at birth as female, PAC members adjusted their approach to recruit male participants. This included specifically connecting with friends, colleagues or other contacts who were male, but also included increased recruitment efforts where young men may be more common (e.g., organized sports). This strategy was used wherever gaps were noted.

Recruited interview participants were mostly female (*n* = 14) and White identifying (*n* = 16). Identified by PAC members as particularly important, participants who had been diagnosed with T1D as both children (< 15 years, *n* = 10) and older teens and adults (≥ 15 years, *n* = 9) were recruited. Further, care was taken to ensure that the interview sample included both young, young adults (< 24 years, *n* = 6) and older, young adults (≥ 24 years, *n* = 13).

## 4 Discussion

Over just a few months, the PAC and the research team recruited 19 young adults with T1D to participate in one‐on‐one interviews, four young adults with T1D, four family and friends and four health professionals and researchers for co‐design events. The efficient recruitment of this sample provided invaluable insights into the nuanced challenges experienced by young adults with T1D, the individual skills developed in response to mental health challenges and the ways that this understanding can shape the development of a structured and integrated mental health programme.

Although the recruitment approach employed was successful in ensuring a relatively swift recruitment process, the paramount focus was on obtaining a co‐design sample that adequately represented the user base anticipated for the developing intervention. Despite a highly engaged PAC and significant effort invested to recruit a sample of young adults living with T1D that was demographically diverse, the recruitment process encountered certain limitations. Notably, this included an overrepresentation of women‐identifying and White participants, indicating potential gaps in inclusivity and representation. This is because while there is evidence that describes differences between sexes and genders in T1D epidemiology (e.g., glycemic targets, diabetes‐related complications) [38, 39], there are gaps in understanding specific to how gender, culture and ethnicity influence and shape the experience of young adults during this highly transitional age stage. These factors are particularly important to understand when we consider their potential to accentuate challenges in mental health and wellness during young adulthood (e.g., self‐agency, relationships, body image) [40, 41] and evidence that has identified their influence on health behaviour (e.g., gender on mental health seeking behaviour) [42]. The intentional effort to recruit individuals from diverse geographic regions and age groups partially addressed these concerns, yet sustained strategies are needed to ensure a balanced and comprehensive participant profile in the second feasibility trial phase of this research. This could include outreach with diabetes groups that service specific groups (e.g., the South Asian chapter of Diabetes Canada) and ensuring that there are adequate resources (e.g., timeline) to do this recruitment. Our recruitment approach highlights the need for resources, including sufficient time and funding, to recruit participants who reflect the population in the co‐design of integrated interventions.

Evidence has demonstrated not only the prevalence of mental health challenges in young adults with T1D but also the complications that can result from its presence, including challenges meeting glycemic targets [37, 41, 43]. Integrated care models like the TECC‐T1D3 programme are complex interventions that seek to integrate services both within and across professional and clinical boundaries. They are complex not only because of their use of governance, systems and technology to achieve these aims, but additionally because of the complex nature of the individuals that they are designed to serve [44, 45]. Responding to mental health challenges in the design of integrated care models is difficult work, as mental health challenges both possess causal complexity and necessitate innovations in research paradigms [46]. The decision not to screen for mental health challenges like diabetes distress as part of the eligibility criteria, and instead assume its presence in all participants, may be viewed as a radical approach. This departure from traditional eligibility screening methods aimed to decrease stigma, lower the threshold to engage diverse individuals who otherwise may not engage in health research and promote candid discussions about mental health and its intricate relationship with diabetes management. Considering the need for whole‐person, integrated diabetes and mental health care for young adults living with T1D, this was a crucial decision and one that we as a research team likely would not have come to make without the aid of the PAC. However, a potential disadvantage of this assumption is that it may inadvertently exclude individuals experiencing the highest levels of distress, who might most benefit from targeted interventions, thereby limiting the generalizability of the findings to those with varying degrees of mental health challenges. There is a continued need to advance the science of partnered research, including the ways that individuals with lived experience can be engaged in research processes like recruitment.

The wealth of data gathered because of our intentional recruitment effort, including in‐depth interviews and co‐design events with diverse participants with varied experiences from across Canada, has played a pivotal role in shaping the development of TECC‐T1D3. This co‐design work advances understanding of the intentional ways that people with lived experience can be engaged in experience‐based co‐design research and the impact of this approach on integrated care interventions. The approach described in this paper provides a framework for developing other integrated care interventions aimed at addressing complex health challenges through patient‐centred design. By incorporating lived experiences into the research process, this model can inform broader strategies for developing holistic and effective care solutions across diverse health domains. While co‐design research emphasizes collaboration between researchers and participants in the design process, experience‐based co‐design research takes this one step further by prioritizing the direct involvement of individuals with lived experience throughout all stages of the research. In this work, our approach was described as empowering young adults living with T1D and mental health challenges to not only inform the development of the TECC‐T1D3 programme but also to actively shape its trajectory. Without PAC insights, gleaned from their personal journeys navigating the complexities of diabetes management and mental health, key discussions that informed recruitment decisions, and ultimately the data collected, would not have been made. Through the application of experience‐based co‐design principles, these decisions have led to the development of a more relevant integrated programme.

## 5 Conclusion

Recruiting diverse voices to contribute to the development and design of integrated care programmes like TECC‐T1D3 is critical to end‐user uptake and sustainability. The diverse composition of the PAC, mirroring the demographic sought in these co‐design activities, amplified our outreach efforts, fostered a rich understanding of the needs of this population and enhanced the relevance and applicability of this integrated and comprehensive model of care. This approach is adaptable to and adoptable in other contexts, providing a blueprint for researchers aiming to replicate this recruitment approach in diverse settings. In future health services and policy initiatives, prioritizing similar collaborative approaches is essential, recognizing the invaluable contributions of those directly impacted by the health conditions and the ways that these perspectives must inform the health systems and care solutions being designed. As inclusive methodologies and approaches are championed, we acknowledge that engaging people with lived experience both in research and as co‐researchers, particularly engaging those beyond the usual participant profile, not only enriches the research process but also ensures the co‐creation of solutions that resonate with the lived experiences of those navigating complex health systems. This collaborative ethos is pivotal in building resilient, patient‐driven healthcare systems that address the multifaceted needs of our communities. Building on this success, PAC members continue to inform the ongoing research as co‐researchers, including recruitment for the TECC‐T1D3 feasibility trial.

## Author Contributions

**Carly Whitmore:** conceptualization, investigation, writing–original draft, methodology, writing–review and editing, formal analysis, data curation, supervision, resources. **Linxi Mytkolli:** conceptualization, methodology, writing–review and editing, supervision, data curation. **Natalie Mangialardi:** conceptualization, methodology, writing–review and editing, project administration, data curation. **Jasmine Maghera:** methodology, writing–review and editing, data curation. **Adam Rudick:** methodology, writing–review and editing, data curation. **Kitty Shephard:** methodology, writing–review and editing, data curation. **Stephanie Zazzera:** methodology, writing–review and editing, data curation. **Anika Saiva:** writing–review and editing, project administration, data curation, resources. **Tracy McQuire:** conceptualization, investigation, funding acquisition, methodology, writing–review and editing. **Peter Senior:** conceptualization, investigation, funding acquisition, writing–review and editing, methodology, data curation. **Diana Sherifali:** conceptualization, investigation, funding acquisition, methodology, writing–review and editing, data curation. **Peter Selby:** conceptualization, investigation, funding acquisition, methodology, writing–review and editing, supervision, data curation.

## Ethics Statement

Ethics approval was received by the Centre for Addiction and Mental Health ethics review board (#100‐22). All interview and co‐design participants consented to participate in the research. As members of the Partner Advisory Council were co‐researchers in this research, they were not consented to participate.

## Conflicts of Interest

The authors declare no conflicts of interest.

## Data Availability

The data that support the findings of this study are available from the corresponding author upon reasonable request.

## References

[1] D. J. Robinson , K. Hanson , A. B. Jain , et al., “Diabetes and Mental Health,” Canadian Journal of Diabetes 47, no. 4 (2023): 308–344. 37321702 10.1016/j.jcjd.2023.04.009

[2] V. Hagger , C. Hendrieckx , J. Sturt , T. C. Skinner , and J. Speight , “Diabetes Distress Among Adolescents With Type 1 Diabetes: A Systematic Review,” Current Diabetes Reports 16, no. 1 (2016): 9. 26748793 10.1007/s11892-015-0694-2

[3] K. A. Reynolds and V. S. Helgeson , “Children With Diabetes Compared to Peers: Depressed? Distressed? A Meta‐Analytic Review,” Annals of Behavioral Medicine 42, no. 1 (2011): 29–41. 21445720 10.1007/s12160-011-9262-4 PMC3140576

[4] C. Bernstein , M. Stockwell , M. Gallagher , S. Rosenthal , and K. Soren , “Mental Health Issues in Adolescents and Young Adults With Type 1 Diabetes: Prevalence and Impact on Gylcemic Control,” Clinical Pediatrics 52, no. 1 (2012): 10–15. 22988007 10.1177/0009922812459950

[5] P. A. Colton , M. P. Olmsted , D. Daneman , et al., “Eating Disorders in Girls and Women With Type 1 Diabetes: A Longitudinal Study of Prevalence, Onset, Remission, and Recurrence,” Diabetes Care 38 (2015): 1212–1217. 25887359 10.2337/dc14-2646

[6] A. Butwicka , W. Fendler , A. Zalepa , et al., “Psychiatric Disorders and Health‐Related Quality of Life in Children With Type 1 Diabetes,” Psychosomatics 57, no. 2 (2016): 185–193. 26774893 10.1016/j.psym.2015.11.001

[7] B. Johnson , C. Eiser , V. Young , S. Brierley , and S. Heller , “Prevalence of Depression Among Young People With Type 1 Diabetes: A Systematic Review,” Diabetic Medicine 30, no. 2 (2013): 199–208. 22698387 10.1111/j.1464-5491.2012.03721.x

[8] M. Monaghan , V. Helgeson , and D. Wiebe , “Type 1 Diabetes in Young Adulthood,” Current Diabetes Reviews 11, no. 4 (2015): 239–250. 25901502 10.2174/1573399811666150421114957 PMC4526384

[9] P. Bower , P. Wallace , E. Ward , et al., “Improving Recruitment to Health Research in Primary Care,” Family Practice 26, no. 5 (2009): 391–397. 19549623 10.1093/fampra/cmp037

[10] K. R. Bell , F. Hammond , T. Hart , A. K. Bickett , N. R. Temkin , and S. Dikmen , “Participant Recruitment and Retention in Rehabilitation Research,” American Journal of Physical Medicine & Rehabilitation 87, no. 4 (2008): 330–338. 18356624 10.1097/PHM.0b013e318168d092

[11] J. R. Banas , S. Magasi , K. The , and D. E. Victorson , “Recruiting and Retaining People With Disabilities for Qualitative Health Research: Challenges and Solutions,” Qualitative Health Research 29, no. 7 (2019): 1056–1064. 30862260 10.1177/1049732319833361 PMC11487479

[12] S. Magasi , “Negotiating the Social Service Systems: A Vital Yet Frequently Invisible Occupation,” supplement, Occupation, Participation and Health 32, no. 1 (2012): S25–S33. 10.3928/15394492-20110906-03 24650786

[13] S. Fletcher , J. Clarke , S. Sanatkar , et al., “Recruiting to a Randomized Controlled Trial of a Web‐Based Program for People With Type 2 Diabetes and Depression: Lessons Learned at the Intersection of e‐Mental Health and Primary Care,” Journal of Medical Internet Research 21, no. 5 (2019): e12793. 31127718 10.2196/12793 PMC6555119

[14] A. Hughes‐Morley , B. Young , W. Waheed , N. Small , and P. Bower , “Factors Affecting Recruitment into Depression Trials: Systematic Review, Meta‐Synthesis and Conceptual Framework,” Journal of Affective Disorders 172, no. 1 (2015): 274–290. 25451427 10.1016/j.jad.2014.10.005

[15] K. Seymour , “Using Incentives: Encouraging and Recognising Participation in Youth Research,” Youth Studies Australia 31, no. 3 (2012): 51–59.

[16] P. Bate and G. Robert , “Experience‐Based Design: From Redesigning the System Around the Patient to Co‐Designing Services With the Patient,” Quality & Safety in Health Care 15, no. 5 (2006): 307–310. 17074863 10.1136/qshc.2005.016527 PMC2565809

[17] S. Donetto , P. Pierri , V. Tsianakas , and G. Robert , “Experience‐Based Co‐Design and Healthcare Improvement: Realizing Participatory Design in the Public Sector,” Design Journal 18, no. 2 (2015): 227–248.

[18] C. B. Hamilton , A. M. Hoens , S. McQuitty , et al., “Development and Pre‐Testing of the Patient Engagement in Research Scale (PEIRS) to Assess the Quality of Engagement From a Patient Perspective,” PLoS One 13, no. 11 (2018): e0206588. 30383823 10.1371/journal.pone.0206588 PMC6211727

[19] E. M. Perfetto , T. R. Love , E. M. Oehrlein , S. Schoch , S. Schrandt , National Health Council Patient‐Centred Core Impact Set Advisory Committee, “ A Foundation for Patient‐Centered Core Impact Sets: Key Learnings From Past and Existing Approaches,” Patient 16, no. 4 (2023): 293–300. 37204700 10.1007/s40271-023-00630-1

[20] M. M. Gilbert , J. A. Chamberlain , C. R. White , et al., “Controlled Clinical Trial of a Self‐Management Program for People With Mental Illness in an Adult Mental Health Service—The Optimal Health Program (OHP),” Australian Health Review 36, no. 1 (2012): 1–7. 22513012 10.1071/AH11008

[21] C. F. Ski , D. R. Thompson , and D. J. Castle , “Trialling of an Optimal Health Programme (OHP) Across Chronic Disease,” Trials 17, no. 445 (2016): 445. 27612634 10.1186/s13063-016-1560-5 PMC5018188

[22] C. L. O'Brien , P. Apputhurai , S. R. Knowles , et al., “Initial Evaluation of the Optimal Health Program for People With Diabetes: 12‐Month Outcomes of a Randomized Controlled Trial,” Psychology and Health 39, no. 3 (2022): 358–378, 10.1080/08870446.2022.2060507. 35465777

[23] P. P. Valentijn , S. M. Schepman , W. Opheij , and M. A. Bruijnzeels , “Understanding Integrated Care: A Comprehensive Conceptual Framework Based on the Integrative Functions of Primary Care,” International Journal of Integrated Care 22, no. 13 (2013): e010, 10.5334/ijic.886. PMC3653278 23687482

[24] P. Selby , L. Vojtila , I. Ashfaq , et al., “Technology‐Enabled Collaborative Care for Youth With Early Psychosis: A Protocol for a Feasibility Study to Improve Physical Health Behaviours,” Early Intervention in Psychiatry 15, no. 4 (2021): 828–836. 32748501 10.1111/eip.13018

[25] O. Melamed , A. Voineskos , L. Vojtila , et al., “Technology‐Enabled Collaborative Care for Youth With Early Psychosis: Results of a Feasibility Study to Improve Physical Health Behaviours,” Early Intervention in Psychiatry 16, no. 10 (2022): 1143–1151. 35103380 10.1111/eip.13266

[26] I. Nielssen , M. Santana , S. Pokharel , et al., “Operationalizing the Principles of Patient Engagement Through a Patient Advisory Council: Lessons and Recommendations,” Health Expectations 27, no. 1 (2023): e13909. 37942678 10.1111/hex.13909 PMC10726262

[27] D. Banner , M. Bains , S. Carroll , et al., “Patient and Public Engagement in Integrated Knowledge Translation Research: Are We There Yet?” Research Involvement and Engagement 5, no. 8 (2019): 8. 30805202 10.1186/s40900-019-0139-1 PMC6373045

[28] T. Greenhalgh , L. Hinton , T. Finlay , et al., “Frameworks for Supporting Patient and Public Involvement in Research: Systematic Review and Co‐Design Pilot,” Health Expectations 22, no. 4 (2019): 785–801. 31012259 10.1111/hex.12888 PMC6737756

[29] E. Manafo , L. Petermann , P. Mason‐Lai , et al., “Patient Engagement in Canada: A Scoping Review of the ‘How’ and ‘What’ of Patient Engagement in Health Research,” Health Research Policy and Systems 16, no. 5 (2018): 5. 29415734 10.1186/s12961-018-0282-4 PMC5804082

[30] M. Bird , M. McGillion , E. M. Chambers , et al., “A Generative Co‐Design Framework for Healthcare Innovation: Development and Application of an End‐User Engagement Framework,” Research Involvement and Engagement 7, no. 12 (2021): 12. 33648588 10.1186/s40900-021-00252-7 PMC7923456

[31] M. Bird , C. Ouellette , C. Whitmore , et al., “Preparing for Patient Partnership: A Scoping Review of Patient Partner Engagement and Evaluation in Research,” Health Expectations 23, no. 3 (2020): 523–539. 32157777 10.1111/hex.13040 PMC7321722

[32] Y. Bombard , G. R. Baker , E. Orlando , et al., “Engaging Patients to Improve Quality of Care: A Systematic Review,” Implementation Science 13, no. 1 (2018): 98. 30045735 10.1186/s13012-018-0784-z PMC6060529

[33] B. Newman , K. Joseph , A. Chauhan , et al., “Do Patient Engagement Interventions Work for All Patients? A Systematic Review and Realist Synthesis of Interventions to Enhance Patient Safety,” Health Expectations 24, no. 6 (2021): 1905–1923. 34432339 10.1111/hex.13343 PMC8628590

[34] “Strategy for Patient‐Oriented Research,” Canadian Institutes of Health Research, 2022, https://cihr-irsc.gc.ca/e/41204.html.

[35] L. Kimball , “Liberating Structures: A New Pattern Language for Engagement,” Systems Thinker 23, no. 1 (2011): 8.

[36] W. H. Polonsky , L. Fisher , J. Earles , et al., “Assessing Psychosocial Distress in Diabetes,” Diabetes Care 28, no. 3 (2005): 626–631. 15735199 10.2337/diacare.28.3.626

[37] L. Fisher , D. M. Hessler , W. H. Polonsky , and J. Mullan , “When Is Diabetes Distress Clinically Meaningful?” Diabetes Care 35 (2012): 259–264. 22228744 10.2337/dc11-1572 PMC3263871

[38] R. R. Huxley , S. A. Peters , G. D. Mishra , and M. Woodward , “Risk of All‐Cause Mortality and Vascular Events in Women Versus Men With Type 1 Diabetes: A Systematic Review and Meta‐Analysis,” Lancet Diabetes & Endocrinology 3, no. 3 (2015): 198–206. 25660575 10.1016/S2213-8587(14)70248-7

[39] M. I. Maiorino , G. Bellastella , O. Casciano , et al., “Gender‐Differences in Glycemic Control and Diabetes Related Factors in Young Adults With Type 1 Diabetes: Results From the METRO Study,” Endocrine 61 (2018): 240–247. 29455365 10.1007/s12020-018-1549-9

[40] L. Lašaitė , R. Dobrovolskienė , E. Danytė , et al., “Diabetes Distress in Males and Females With Type 1 Diabetes in Adolescence and Emerging Adulthood,” Journal of Diabetes and its Complications 30, no. 8 (2016): 1500–1505. 27613444 10.1016/j.jdiacomp.2016.08.013

[41] L. Lašaitė , R. Ostrauskas , R. Žalinkevičius , N. Jurgevičienė , and L. Radzevičienė , “Diabetes Distress in Adult Type 1 Diabetes Mellitus Men and Women With Disease Onset in Childhood and in Adulthood,” Journal of Diabetes and its Complications 30, no. 1 (2016): 133–137. 26490756 10.1016/j.jdiacomp.2015.09.012

[42] I. Sagar‐Ouriaghli , E. Godfrey , L. Bridge , L. Meade , and J. S. L. Brown , “Improving Mental Health Service Utilization Among Men: A Systematic Review and Synthesis of Behavior Change Techniques Within Interventions Targeting Help‐Seeking,” American Journal of Men's Health 13, no. 3 (2019): 1557988319857009. 10.1177/1557988319857009 PMC6560805 31184251

[43] L. Fisher , W. H. Polonsky , D. M. Hessler , et al., “Understanding the Sources of Diabetes Distress in Adults With Type 1 Diabetes,” Journal of Diabetes and its Complications 29, no. 4 (2015): 572–577. 25765489 10.1016/j.jdiacomp.2015.01.012 PMC4414881

[44] C. Steele Gray and J. Shaw , “From Summative to Developmental: Incorporating Design‐Thinking into Evaluations of Complex Interventions,” Journal of Integrated Care 27, no. 3 (2019): 241–248.

[45] C. Steele Gray , A. Grudniewicz , A. Armas , J. Mold , J. Im , and P. Boeckxstaens , “Goal‐Oriented Care: A Catalyst for Person‐Centred System Integration,” International Journal of Integrated Care 20, no. 4 (2020): 8. 10.5334/ijic.5520 PMC7646288 33199976

[46] K. Bhui and A. Cipriani , “Understanding and Responding to the Drivers of Inequalities in Mental Health,” BMJ Mental Health 26 (2023): e300921. 10.1136/bmjment-2023-300921 PMC10749028 38114130

